# Gut microbiota and colorectal cancer

**DOI:** 10.1007/s10096-016-2881-8

**Published:** 2017-01-07

**Authors:** R. Gao, Z. Gao, L. Huang, H. Qin

**Affiliations:** Tongji University School of Medicine affiliated Tenth People’s Hospital, No.301 Middle Yanchang Road, Shanghai, 200072 China

## Abstract

The gut microbiota is considered as a forgotten organ in human health and disease. It maintains gut homeostasis by various complex mechanisms. However, disruption of the gut microbiota has been confirmed to be related to gastrointestinal diseases such as colorectal cancer, as well as remote organs in many studies. Colorectal cancer is a multi-factorial and multi-stage involved disorder. The role for microorganisms that initiate and facilitate the process of colorectal cancer has become clear. The candidate pathogens have been identified by culture and next sequencing technology. Persuasive models have also been proposed to illustrate the complicated and dynamic time and spatial change in the carcinogenesis. Related key molecules have also been investigated to demonstrate the pathways crucial for the development of colorectal cancer. In addition, risk factors that contribute to the tumorigenesis can also be modulated to decrease the susceptibility for certain population. In addition, the results of basic studies have also translated to clinical application, which displayed a critical value for the diagnosis and therapy of colorectal cancer. In this review, we not only emphasize the exploration of the mechanisms, but also potential clinical practice implication in this microbiota era.

## Introduction

Colorectal cancer (CRC) has been a common malignancy in the world, especially in China, in recent years. According to the epidemiological data, the 5-year prevalence proportion has reached 74.6 and 58.3 per 100,000 in men and women respectively [1]. An updated estimation reveals that more than 376, 000 new cases of CRC and 191,000 deaths occur every year in China [2]. CRC has long been investigated and it is classified into two typical types: colitis-associated colorectal cancer (CAC) and sporadic colorectal cancer (SCC), according to genomic mutation diversity. Hereditary syndrome has been identified with a total of fourteen mutations [3]. The inner involved signal pathways are totally different between these two relatively independent phenotypes, but they also share a few sequential genetic mutations. CAC is always associated with inflammatory bowel disease, an inflamed disorder phenotype in the young population. SCC is usually used to refer to the common colorectal cancer that considered without family heredity. CRC is a malignant disease which involves multiple factors during its multi-stage development. The initiating events of CRC have been proved to be APC mutation in SCC and TP53 mutation in CAC. The etiology of CRC has been investigated using large cohorts and confirmed by animal models, and the consensus conclusion contains genetic background and environmental risk factors such as diabetes, cholecystectomy, obesity, high fat diet, and processed and red meat [4–9]. However, a large number of studies have recently reported that the gut microbiota may also participate as an essential contributor factor in the initiation and development of CRC. Here, we will focus on the potentially plausible relationship between gut microbiota and colorectal cancer.

### Overview of microbiota in the gut

The gut contains a complicated environment that is settled by bacteria, fungi, and viruses. The total number may reach 100 trillion, and the number of microbe cells is estimated to be 10-fold more than the human cells. This densely resident microbial community consistently communicates with the host and also enhances the epithelial defense against pathogens, accelerates the maturity of the immune system, and absorbs the nutrition from ingested foods [10, 11]. Despite the mucus layer, which consists of various macromolecules and secreted antimicrobial molecular and intercellular tight connection proteins, the gut microbiota also possess the capacity to defend pathogens by inducing IgG antibodies through recognition of their conserved antigen part of gram-negative bacteria [12, 13]. The gut microbiota not only protect the local homeostasis, but also mediate the related organ. For example, an in-vivo experiment proved that the gut microbiota was manipulated by intestinal lectins to decrease alcohol-associated steatohepatitis [14]. Along with the evolution of gut microbiota, body cells also demonstrate effective pathways for avoiding the pathogen infection. *Salmonella Typhi* is a well-known pathogen that once caused great damage to human health. Recently, Spano and his colleagues proposed a neo-mechanism, Rab32-dependent cell autonomous antimicrobial approach, which was critical for the host to restrict *Salmonella Typhi* infection [15]. Gut microbiota residing in infants is derived from the obstetric canal and mother’s skin after birth, then becomes matured and keeps relatively stable during a long time of lifespan and changes in the elderly time. Despite age, a variety of factors such as diet, drugs, sports, and genotype also impact the gut microbial community [16–20]. In a healthy gut, the dominant core bacteria are composed of Firmucutes, Proteobacteria, Bacteroidetes and Actinobacteria at the phylum level. However, the gut micro-community displays a diverse structure at the genus and species levels.

### Gut microbiota and colorectal cancer

With the strong evidence displayed by multi-direction proofs, the stomach cancer associated pathogenetic bacterium (*Helicobactor pylori*) has been identified and recognized internationally as the level one carcinogen. Likewise, with a more complicated microbial community covering the inner surface of the colon, this earlier discovery enlightens the researchers to seek for a similar pathogen to explain the initiation and development of CRC. To explore the possible role of microbiota in the etiology of CRC, the researchers first separate and culture several bacteria in various media. However, less supportive evidence can illustrate the role of microbiota in the CRC development. Along with an improvement in detection technology, more and more studies utilize the next sequencing technology to explore the candidate carcinogenetic pathogen in the gut among population with distinct differing disease phenotypes.

The first report that links the gut microbiota with CRC is published by Weisburger and his colleagues [21]. Later, more and more studies confirm the relationships between pathogenetic bacteria and colorectal cancer. For example, infection with *Streptococcus bovis*, a group of gram-positive cocci, has been reported to be a risky sign for colon tumors [22]. Kostic and his team identify high enrichment of Fusobacteria sequence in colorectal carcinoma tissue using whole genome sequencing, and confirm the result in a large scale study of colorectal cancer tissue samples [23]. Similarly, Enterotoxigenic *Bacteroides fragilis* and *Fusobacterium nucletum* are identified to be highly expressed in colorectal cancer tissue compared to the matched tissue, and *Fusobacterium nucletum* is proved to be associated with high microsatellite instability [24]. Our previous study also identifies a discrepancy in tissue-associated gut microbiota between colorectal cancer patients and healthy volunteers [25]. In addition, mucosa-associated *E.coli* belonging to the B2 phylogroup is found to be more prevalent in CRC tissues, and is identified to encode cyclomodulin which is vital for colon epithelia cell mutation [26].To explore subsequently, *Fusobacterium nucleatum*, which belongs to Fusobacteria, has been isolated from tumor tissue and proved to be invasive in the in-vitro experiments. In addition, *Fusobacterium nucleatum* also has a positive correlation with lymph node metastasis in CRC [27].Furthermore, Zhao and his colleagues study the stool samples of CRC patients in China, and found find that *Bacteroides fragilis*, *Enterococcus*, *Escherichia/Shigella*, *Klebsiella*, *Streptococcus*, and *Peptostreptococcus* display a higher relative abundance in CRC patients, while *Roseburia*- and Lachnospiraceae-related OTUs dominat a high load in the healthy controls [28]. In another study, researchers also compare stool samples and find that the CRC patients have a lower microbiota diversity and *Clostridia* abundance, but a high abundance of *Fusobacterium* and *Porphyromonas* at genus level [29]. The lumen and tissue microbiota are obviously different in microbial structure. In the tissue samples, beneficial microbes such as *Bifidobacterium*, *Faecalibacterium*, and *Blautia* were are significantly reduced, while *Fusobacterium* is enriched in the CRC patients [30]. However, the stool samples show a significant different microbial landscape with *Paraprevotella*, *Eubacterium*, and several other bacteria enriched in CRC patients [30]. Inflammation is also an important factor that contributed to CRC progress via gut microbiota. Arthur finds that *E.coli* NC101 will increase the colon tumor load in AOM treated IL10^-/-^ mice. When he deletes the polyketide synthase (*pks*) Genotoxic Island in *E.coli* NC101, a significant decrease of tumor load and invasion capacity are observed [31]. Clinical study also revealeds a close connection between *E.coli* and advanced stages, and animal experiment shows a high tumor load under incubation with *E.coli* [32, 33]. To better understand adenoma-carcinoma sequence-related gut microbiota and functional genes, sequential continuous detection is performed in the stool samples. By metagenomic analysis, the researchers find that a total of 130,000 genes are different in any two-group comparison among the healthy, adenoma, and CRC patients [34]. And further analysis including the diet pattern concludes that fruit and vegetable consumption are related to the healthy group, while high level of red meat consumption and C-reation protein are associated with the carcinoma phenotype. In addition to these findings, the study also show that sugar transporter and a couple of amino acids consist of histidine, lysine, methionine, cysteine, and leucine are enriched in the healthy when compared with adenoma or in adenoma in comparison with carcinoma patients. Despite the stool microbiota change, the architecture of gut microbiota is also altered in the tissue samples by the sequencing. By 16S ribosomal RNA sequencing, researchers identify that *Fusobacterium*, *Parvimonas*, *Gemella*, and *Leptotrichia* are enriched and anti-inflammatory *F. prausnitzii* loses its abundance in early-stage colorectal cancer [35]. Furthermore, current studies also demonstrates that the *Fusobacterium nucletum* is strongly associated with CpG island methylator phenotype [36]. A recent study explores the gut microbiota in matched tissue and stool samples, host genes, and immune system together. The results show that firstly the fecal microbiota only has partial similarity with the tissue microbiota. Then a new cluster set is proposed and named co-abundance groups (CAGs) which is similar to enterotypes, and identified decreased Bacteroidetes cluster 1 and Firmicutes cluster 1, also cluster 2 of Bacteroidetes and Firmicutes as well as pathogen and *Prevotella* cluster in the colorectal cancer tissue community [37]. The study also identifies that CAGs are also associated with human immune responses such as IL17a, myc, and STAT3.

To illustrate the relationships between CRC and gut microbiota, several typical rodent models which simulate the CRC development are also performed. In a dimethylhydrazine-induced model, a obvious separated lumen gut microbiota is observed [38]. APC^min/+^ mice raised in a germ-free environment display a reduced tumor load after that in the SPF condition [39]. When the germ-free mice are delivered with gut microbiota from tumor burden mice, they display more and larger tumors. To verify that the increased tumor burden that appears in germ-free mice are derived from the harmful microbiota, antibiotics are applied to the receptor mice which, as a result, did slow down the carcinogenesis process [40]. These experiments show us the critical role of gut microbiota in colorectal cancer and also plausible causality of gut microbiota for the rodent models. However, gut microbiota in the rodent models differ significantly from the human beings, so it is not certain whether the same ideal results will re-emerge in the human-derived gut microbiota. Nielson and his partners transplant human donor stool into the mice, and results show that the tumor burden is apparently associated with the gut microbiota structure at baseline in the germ-free mice [41]. These results sufficiently confirm that dysbiosis in the gut is one of the reasons that caused colorectal cancer.

### Pathogen identification by the immune system

**Fig. 1:**
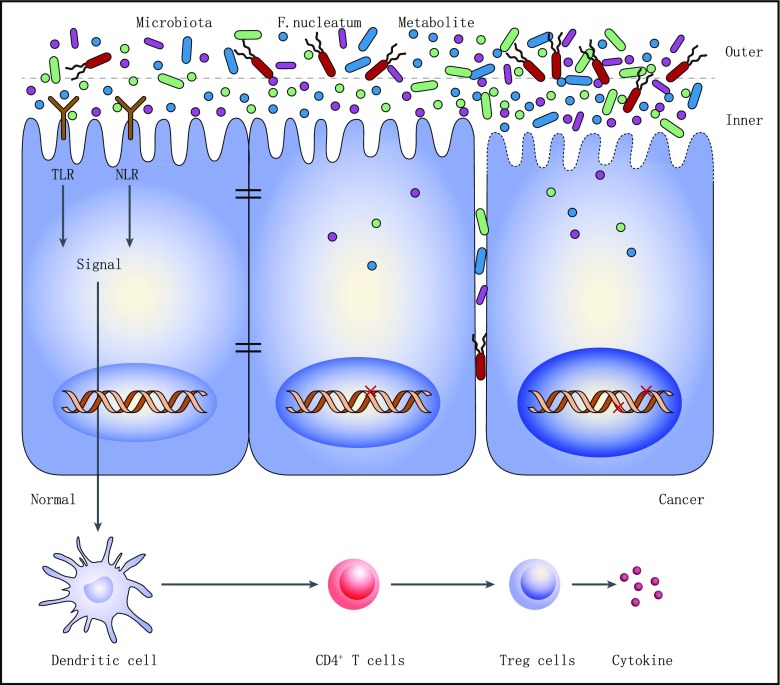
The interaction between host and commensal microbes in the gut. Under normal conditions, the signals from commensal bacteria could be detected by sensors TLR and NLR. Signals are passed down to trigger the immune system activation and cytokine release to maintain the balance. When the microenvironment changes, the pathogens (*Fusobacterium nucleatum*, etc.) pass through the inner mucus layer and invade into the stroma by destroying the tight junction and inducing drastic responses

When ingested microorganisms reach the gut, it is of vital importance for the immune system to identify them to protect the host (Fig.1). Currently, several receptors have been recognized to mediate the process. The host possesses immune innate receptors named pattern recognition receptors (PRRs) to search the pathogen-associated molecular patterns (PAMPs) that expressed in the pathogens. We will describe and discuss these receptor-pathogen interactions based on achievements so far.

**Fig. 2:**
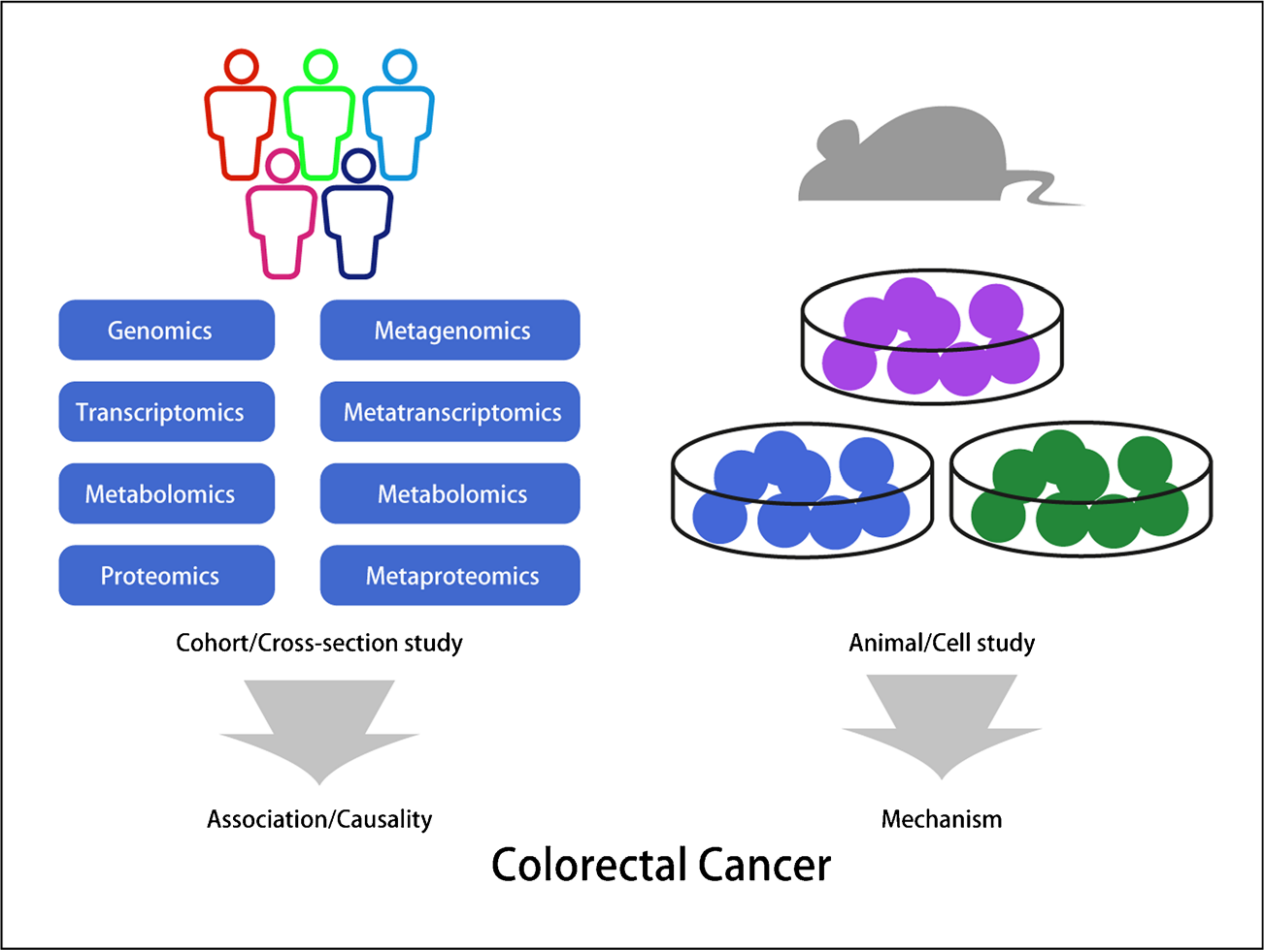
A variety of factors affect the gut microbiota and host health. The relationship between gut microbiota and host is complicated, with direct and indirect effects. To study the relationship, it is important to take the multi-omics study into consideration both in the host and the microbiota (cohort and cross-section studies). In addition, the model animal and cell studies would provide another aspect of targeting mechanisms. All the factors taken together would contribute a comprehensive understanding of the complicated and sophisticated relationship between gut microbiota and the occurrence and development of colorectal cancer

### Toll-like receptors

Among the PRRs, the Toll-like receptors (TLRs) are studied earliest to detect the signal from pathogens. The nature of TLRs is type I membrane glycoprotein, which belongs to a superfamily that includes interleukin-1 receptors. The structure of membrane varying between them discriminate the TLRs and IL-1R. TLRs do not only locate in the membrane of epithelia cells (TLR1, TLR2, TLR4, TLR5, TLR6), they also express in the endosome membrane (TLR3, TLR7, TLR8, TLR9, TLR11, TLR13) [42].Pathogens always produce lipoproteins to induce the host immune response, trigger monocyte apoptosis, and activate the NF-κB signal pathway via TLR-2 [43]. TLR-2 also acts as a mediator to promote the cell activation by peptidoglycan and recruited by macrophages to recognize the pathogens in in-vivo experiments [44, 45]. Mammalian TLR-3 is identified to recognize the double-stranded RNA which is associated with viral infection [46]. Study on TLR-4 has proved that this protein is involved in the cooperator for CD14 which would activate the lipopolysaccharide-induced NF-κB signaling [47]. By gene knockout mice model, TLR-3 and TLR-4 have been identified to function normally with an essential factor Toll/IL-1 receptor domain-containing adaptor [48]. While TLR-7 and TLR-8 mediate the recognition of species-specific single-stranded RNA from virus [49, 50]. TLR-9 has been proved to help the cellular response to CpG DNA of infectious pathogens [51]. The TLRs has an asymmetrical distribution in human peripheral blood mononuclear cells. According to Hornung’s work, TLR-1 and TLR-6 expresse in all the cells including plasmacytoid dendritic cells (PDC), B cells, NK cells, T cells and also monocytes [52]. TLR-2 is highly expressed in monocytes. TLR-3 has a relative high expression in NK cells and all low in other cells. TLR-4 expresses high in monocytes. TLR-6 is mainly detected in B cells, but can also be detected in NK cells, monocytes, and PDC. TLR-7 is moderately expressed in PDC and B cells. Detected expression of TLR-8 is only high in monocytes. TLR-9 is almost three-fold more highly expressed in PDC than in B cells. However, TLR-10 is expressed highly only in B cells and PDC; the other cells are relatively low. All TLRs activated the MyD-88 dependent pathways to induce the downstream immune responses [53].

To better understand the mechanism of toll-like receptor signaling, Akira summarizes the pathways that are involved in detail to show us a more visible landscape [54]. In conclusion, after binding to the TLR/IL-1R, signals pass down to trigger a cascade. The essential molecules included during this process may include adaptor molecule myeloid differentiation primary-response protein 88 (MyD88), transforming growth factor-β activated kinase, and tumour-necrosis factor receptor-associated factor 6 [54]. Despite the direct killing functions, the activated TLRs in the macrophages also induce the increased expression of vitamin D receptor, and thus facilitate the function of antimicrobial peptide on mycobacterium tuberculosis [55].

### NOD-like receptors

The innate immune system provides a rapid response to the pathogens without a memory process. Such a process relies on the perception of conserved microbial motifs that named PAMPs as described before. After acquiring the signals, the host launches a series of defensive mechanisms against the pathogens. Apart from the TLRs in the cell membrane, another defense system, NOD-like receptors, has also been identified by *Shigella flexneri* infection [56]. NOD stands for nucleotide-binding oligomerization domain inside the cells. The NOD-like receptors (NLRs) are essential for defensive architecture against invasive bacteria and the bacteria products in the inner space of cells. In addition, the NLRs also show penetrating sensor of the “danger signals” from injured or dying cells. The already known NLRs include NOD1-5, NALP1-14, CII TA, Ipaf, and Naip, which constitute a big family [57]. Among them, the first three proteins have been investigated and are well understood. The structure of NLRs consist of various domains such as leucine-rich repeat domain in the C-terminal, nucleotide-binding domain in the center part, and a protein–protein interaction domain in the N-terminal [57]. According to the N-terminal domains, NLRs have been clustered into three groups: caspase recruitment domain containing NODs, pyrin containing NALPs, and baculovirus inhibitor repeat containing NAIPs [58]. These NLRs sense the PAMPs and subsequently trigger conformational rearrangements to conduct the signal spread and finally activate the diverse signal pathways. For example, *Salmonella* has shown the ability to inhibit the expression of NLR Family CARD domain containing protein 4, thus not only decreasing the secretion of IL-1b, but also preventing the apoptosis of B cells, maintaining the ideal niche for its persistence and reproduction [59].

NOD1 can recognize the g-D-glutamyl-meso-dia-minopimelic acid which exists in Gram-positive and Gram-negative bacteria. It prevents the candidate pathogens by innate immune functions. In addition to that, the NOD1 also shows the potential possibility to hamper the process from colon inflammation to tumorigenesis. NOD2 has been showed to respond to a wide variety of bacteria. The inside type III secretion system cooperates with this function of *Salmonella*. Knockout of *NOD2* gene in the mice displays an increased gut load of bacteria, decreased capacity to prevent the colonization of pathogens and also damaged crypt function. The mutation of *NOD2* also triggers inflammatory diseases [60]. Double knockout of *NOD1* and *NOD2* causes increased gut permeability, decreased expression of E-cadherin, and impaired antimicrobial function in the C57BL/6 mice model [61]. NOD2 holds great importance for the balance in the intestinal microbiota [62]. Nevertheless, a recent study shows that though NOD1-deficient mice has a weaker epithelial barrier in the gut, the microbiota composition does not change. The NOD2-deficient-mice show more interesting results, among which no significant alterations are observed in immune damage and microbiota profile [63]. In addition, the microbiota change is associated with the housing conditions of the mice. This result needs to be confirmed by more similar experiments.

The other NLRs involved mechanisms also have been overviewed and summarized with the target of NLRP3, NLRC4, NAIP, and NLRP1 [64]. It was revealed that mitochondria plays an important role in the activation of NLRP3 inflammasome. The mitochondria provides a convenient platform for NLPR3 to assemble, and also effectors such as a mitochondrial reactive oxygen species which derived from itself to activate the immune process. Another two regulators, guanylate-binding protein 5 and double-stranded RNA-dependent protein kinase, may also contribute to this process. The NLRC4 inflammasome activates the bacteria secretion system, type III and IV, through detecting the bacteria proteins. NAIPs will be activated by binding to the bacteria flagellin or type III system. Lethal toxin may be the activator for NLRP1 inflammasome, and it has also been shown that NLRP1 is associated with the viral immune responses and proteolytic function [64]. NRPL10 has been shown to be crucial to maintain adaptive immunity, and NRPL12 knockout mice show a susceptibility to colitis and colon tumorgenesis, which display its important role in the gut homeostasis [65, 66].

### Hypothesis models associated with colorectal cancer

To better understand the role of gut microbiota in the initiation and progression of colorectal cancer, a few hypotheses are raised by the researchers. The driver–passenger model is set in an totally different respect, with the intention of illustrating the various roles of commensal bacteria in the development of colorectal cancer [67]. This model classifies microbes into two different groups, and shows that the driver microorganisms cause DNA damage in epithelial cells which may start the progression of CRC in the first-time spatial location, then the tumor microenviroment subsequently changes to favor the blooming of passenger bacteria which may dominate in the tumor site later. This model highlights the point that although the driver bacteria initiate colorectal cancer, these microorganisms will not always exist as a marker, resembling genetic mutation, in the surroundings of tumor cells, but will disappear from the cancerous tissue as a loss of growth advantage. This model may be effective to illustrate the discrepancy among various results in different studies, but it also increases the challenges regarding what we can do to clarify this ambiguous relationship, and how.

The other model proposed is the keystone hypothesis [68]. This model declares that a keystone pathogen is defined as a microorganism that supporting the disease-associated dysbiotic microbiota. The microorganism may display a relatively low abundance in the ecosystem. Here, this theory no longer emphasizes the strength level in the disease-related microbiota, but the functions that contribute to and maintain the imbalanced state. The base of this hypothesis is the pathogenesis of *Porphyromonas gingivalis* and Periodontitis. In a model, the *Porphyromonas gingivalis* could still induce periodontitis even at very low abundance (less than 1%) in the whole community. In addition, the accompanying microbiota also change to fire the inflammation. If the pathogen is erased in a condition, no periodontitis would occur even with the same commensal bacteria. With a similar thought process, *Klebsiella* *pneumonia* and *Proteus mirabilis* could be treated as the keystone pathogens in inflammatory bowel disease, as well as the role of enterotoxigenic Bacteroides fragilis in colon cancer. This hypothesis provides a new insight for us to reconsider the potential role of gut pathogens in the initiation and development of related disorders. However, logistic identification is still required to confirm this notion with more model experiments.

Garrett and his colleagues propose their theoretical models of microbe or microbial community carcinogenesis on colorectal cancer [69]. One model is the specific microorganism, the second model is the microbial community, and the last is the sequential collaboration by the single and community.

Since current studies have identified several candidate pathogenic bacteria that have a close relationship with colorectal cancer, the involved mechanisms also need to be investigated. The first model is well understood and has been investigated by many researchers. The community model may well explain the current understandings in inflammatory bowel disease.

### Carcinogenesis mechanisms of candidate species

The clinical abnormal distribution of microbiota in colorectal cancer and matched normal tissue has been described in the former context. Here, we will discuss mainly the mechanism of main candidate species based on current results.

### Fusobacterium nucleatum

Studies of the relationship between *Fusobacterium nucleatum* and colorectal cancer has been reported extensively. Currently, the researchers have started to focus on the potential mechanisms of the pathogen. A recent study reveals that CRC cell proliferation is stimulated through FadA binding to E-cadherin expressed in the CRC cells including HCT116, DLD1, SW480 and HT29 [70]. The APC^min/+^ mice model demonstrates that more colon tumors exist in mice fed with *Fusobacterium nucletum* in comparison with *streptococcus* [71]. In addition, *Fusobacterium nucletum* does not induce colitis or accelerate the colitis-associated cancer. However, it is able to recruit the immune cells and hence provides a proinflammatory microenvironment for the initiation and development of colorectal cancer. Isolation from the inflamed tissue shows that the *Fusobacterium nucletum* diaplays a high invasive ability and evokes a high expression of MUC2, as well as tumor necrosis factor alpha in both in-vivo and in-vitro studies [72]. Study has also shown that *Fusobacterium nucleatum* inhibits the NK cell function by binding to the inhibitory receptor TIGIT via its Fap2 protein [73]. A trap which needs to be avoided here is that not all the isolated *Fusobacterium nucletum* exerts the same pathogenic ability. In fact, based on current evidence, isolates of *Fusobacterium nucletum* from the inflamed parts are more invasive than the normal tissue either from the IBD patients or the healthy controls [72, 74]. The mechanisms may be due to the copy number variation among the bacteria strains [75].

### ETBF

Cumulative evidence has proposed a gut pathogenic bacteria-enterotoxigenic *Bacteroides Fragilis* (ETBF), which encoded B. fragilis metalloprotease toxin (BFT) to induce diarrhea in most reports, as a carcinogenic bacteria in colorectal cancer [76]. The clinical proofs that point out the association with colorectal cancer have been described before. Previous knowledge on the function of ETBF is mainly focused on its potential to remodel the epithelial cytoskeleton and F-actin structure by targeting the E-cadherin [77]. Here, we will mainly discuss the involved mechanisms, based on several current discoveries. ETBF has been shown to trigger colitis and colonic tumors in multiple intestinal neoplasia mice [78–80]. This bacteria will induce activation of transcription-3 (Stat3) pathway with characterization of T helper type 17 response [81]. In addition, BFT has been demonstrated to trigger cell proliferation and activate c-Myc expression in vitro, as well as increase the polyamine metabolism and induce DNA damage [82, 83].

### E.coli

*Escherichia coli* (E.coli), a Gram-negative, anaerobic commensal bacteria, is common in the gut microenvironment. Several studies have linked the E.coli with colorectal cancer risk. Nevertheless, the involved mechanism is still unknown. Clinical study shows that cyclomodulin-producing E. coli colonizes in most cancerous samples [26, 84]. Isolates of *E.coli* from colon cancer tissue show adherence and invasive potential to a specific cell line and also induce interleukin-8 expression [85, 86]. Currently, the *pks* island containing *E.coli* has been shown to express Colibactin gene and induce DNA damage, chromosome aberrations, as well as increased gene mutations in vivo [87]. The increased gene mutation may due to the depletion of the DNA mismatch repair system related to the effector protein of *E.coli* [88]. A colibactin warhead that directly binds to the duplex DNA with spirobicyclic structure provided more clear evidence for its potential role with regard to carcinogenesis [89]. The *pks* positive *E.coli* is first isolated from inflammatory disease; however, a recent finding also identifies that the *E.coli* also contained this pathogenic island [90]. This finding may provide new evidence that links inflammatory disorders with colorectal cancer. In addition, in-vivo study also demonstrates that enteropathogenic *Escherichia coli* promotes cancer cell survival by induction of macrophage inhibitory cytokine 1, thus activating the transforming growth factor β-activated kinase 1 and RhoA GTPase following pathogen infection, and also the continuous expression of COX-2 [91, 92]. When the Caco-2 cell is co-cultured with *E.coli* in vivo, genes that correlate with the oxidation stress are up-expressed, suggesting a defensive response which may be due to the changed microenvironment in the system [93]. Other candidate carcinogenetic microorganisms also include *streptococcus bovis*, *H. pylori*, and *Clostridium*, which have been described in detail elsewhere [21].

Alteration of gut microbiota may also induce cytokine imbalance. The IL-17 family from the Th17 cell has been found to be closely associated with colorectal cancer. IL-17A, IL-17 F, and IL-22 are identified to promote the tumorigenesis of colorectal cancer in early studies. Then IL-17C is also proved to be required in the formation of colorectal cancer in gene knockdown mice model [94]. The detailed mechanism may due to the effect of prolonged epithelial cells by induction of BCL-x_L_ and BCL-2 expression [94].

Other studies of the mechanism show that activating of EGFR-MAPK pathway is also a risk path in colon cancer progress [95]. The ingested alcohol is transformed to high level of acetaldehyde in the colon, but this effect is prevented with antibiotics that target the gut microbiota [96]. The experiment suggests that the gut microbiota promote colon cancer by targeting the metabolites. Short-chain fatty acids decrease in colorectal cancer patients, but if the concentration is increased, it stimulates the epithelia metabolism, decreases the intracellular O_2_ load, and protectes the tight barrier function [97].

### Beneficial effects of Faecalibacterium prausnitzii

In the previous part of this paper, we summarize the candidate gut microorganisms which relate to colorectal cancer. In fact, the researches not only identify the pathogens, but also the beneficial microorganisms which can theoretically be treated as probiotics. As a member of the Clostridium leptum group, *Faecalibacterium prausnitzii* could represent the beneficial commensal bacteria. Clinical investigation has found that the bacteria is at low abundance in ulcerative colitis patients [98]. However, the ability of this potential mechanism in terms of anti-inflammatory and colitis prevention may depend on the capacity to induce IL-10 secretion and Treg cell modulation. The cytology experiment reveals that *Faecalibacterium prausnitzii* could upregulate the ovalbumin-specific T-cell proliferation and reduce the number of IFN-γ + T cells to yield anti-inflammatory effects [99]. Recently, an anti-inflammatory protein of 15 kDa called the microbial anti-inflammatory molecule (MAM) is identified by the researchers. And in the subsequent in-vivo and in-vitro experiments, the MAM shows a significant function in decreasing the NF-KB pathway and also alleviates chemically induced mouse colitis [100]. *Faecalibacterium prausnitzii* has shown a very promising probiotic property as a partner with the gut commensals in inflammatory diseases and colon tumors [101]. This butyrate-producing commensal bacteria is definitely worth more attention and exploration.

### Clinical value of the microbiota

Almost all the candidate pathogenic microbes are identified from clinical samples, and then the carcinogenesis mechanisms were investigated in vivo and in vitro. However, many studies halte at this point. Nevertheless, all the discoveries from basic experiments should be closely connected with the clinical outcome. The current study shows that the gut microbiome can be used as an effective tool in early screening for colorectal cancer. When applied to three groups of patients, the gut microbiome shows a desired ability to distinguish among the adenoma to carcinoma sequences, in combination with several known demographical factors [102]. Another study demonstrates that the effectiveness of the microbiome is analogous with FOBT in detecting colorectal cancer, but greatly increases the sensitivity by ∼45% when the two are in combination [103]. In addition, metagenome analysis not only confirmes the given dysbiosis but also found 20 gene markers during the comparison and identifies four gene markers that validated finally [104]. Genetic mutation has been reported to be related to the prognostic outcome of colorectal cancer. The combination of microsatellite stable or microsatellite instability low, CpG island methylator phenotype positive, BRAF mutation positive, and KRAS mutation negative have been reported to have the worst outcome among various gene mutation groups [105]. In addition, the specific pathogen could also be used as a disease state prediction tool [74]. One study finds that not all colon lesions are related to *Fusobacterium nucletum*, only high-grade dysplasia and colorectal cancer, not adenoma, display a higher expression in colon tissues [106]. This study also identifies the association between *Fusobacterium nucletum* and colorectal clinical outcome, such as patients with a lower abundance of *Fusobacterium nucletum* will have a longer overall survival time. A large cohort displays that the high expression of Fusobacterium nucletum is associated with high microstallite instability, high abundance of *pks* ^+^ E.coli and a bad prognosis, similarly as before, by Cox proportional hazards analysis [24, 107]. The candidate reason for observed poor prognosis may due to the inverse association between *Fusobacterium nucletum* and the infiltrated T-cell amount, which has a negative correlation with cancer, in the cancer sites [108]. *Fusobacterium* abundance in cancerous tissues also shows an association with molecular patterns of colorectal cancer. High expression of *Fusobacterium* has a positive association with CpG island methylator phenotype positivity status, TP53wild-type, hMLH1methylation positivity, and microsatellite instability, as well as CHD7/8 mutation positivity [109].

In addition to the primary prevention role, gut microbiota also show great potential in cancer therapy. In immunotherapy against cancers, specific bacteria of Bacteroidetes have shown better efficacy. Recently, Vetizou and his team find that intake of *Bacteroidetes fragile* or related *Bacteroidetes* or species-specific activated T cells will enhance the treatment outcome, targeting CTLA-4 [110]. Another experiment confirms that commensal *bifidobacteria* also strengthens the anti-tumor effect in the same way as a checkpoint blockade, and almost inhibites tumor growth in combination [111]. The potential mechanism involved targeting the CD8^+^ T cells in the microenvironment. These exciting discoveries highlight the important clinical preventive and therapeutic values of gut microbiota, and initially call for more clinical trials with a larger sample size to confirm the conclusions.

### Manipulation toward the microbiota

Just as described above, the microbiota has been classified into different groups. Like the proverb that the same knife cuts bread and fingers, even the same strains may also function differently under various conditions. So how to manipulation the gut microbiota to a beneficial direction will be an important issue in the management of disease prevention and even therapy. Diet is the most important factor that can be modified and thereby affect the cancer risk through gut microbiota. Diet had a discriminate function on the gut microbiota called enterotypes. Long-term consumption of animal fat has been shown to favour *Bacteroides* enterotype, while carbohydrate favoured the prevotella *Prevotella* enterotype [112]. Dietary intervention is proved to be effective in improving gene diversity and clinical indexes, but have a limited role in inflammation [113]. A lower component of oligosaccharides, disaccharides, monosaccharides, and polyol display an improved micriobiota diversity and bacteria load compared to the control [114]. Study also shows a rapid transition of gut microbiota in response to diet change [115]. The crosstalk mechanism between dietary lipids and gut microbiota is based on the TLR signal pathway [116]. Therefore, manipulation of the gut microbiota by dietary pattern alteration is considered to be an economical, effective, and beneficial method of dampening cancer risk in the susceptible population. An in-vitro study focused on dietary fibers reveales that pH would shift and *Faecalibacterium prausnitzii* dominates at the species level and an improvement created in the diversity of the gut microbiota [117]. The effect of change in exercise pattern on the gut microbiota is also investigated, and shows that exercise significantly improves gut microbiota diversity and the relative abundance of specific microbes [18].

The most ideal method for modifying the gut microbiota may be direct consumption of probiotics. To study the complicated relationships among different microorganisms, research has been carried out on four *Bifidobacteria* strains to evaluate their metabolic functions [118]. *Bifidobacteria* function on the gut microbiota has also been investigated in the murine models by multi-omics. The results show that the relative abundance of Rikenellaceae increases and Lachnospiraceae decreases over time. In the group with combinated *Bifidobacteria*, the relative abundance of Bacteroidaceae is increased at the family level. And the final analysis reveales that the composition and structure of gut microbiota with single probiotics are different from those with two or more bifidobacteria, which is also identified by Wang et al. [119]. Our clinical trial also demonstrates the benefits of probiotics for colorectal cancer patients, including lower infection and short length of stay [120]. In addition, as probiotics can not defend against elimination by the gastric juices, the amount of probiotics that reaches the colon finally to carry out their function is uncertain. So researchers have also proposed that prebiotics would certainly be more ideal to promote a bloom of gut beneficial bacteria. Prebiotics are defined as the non-indigestive components that pass through the gastric and small intestinal parts and stimulate gut beneficial microbiota in the colon and rectal parts [121]. Once probiotics and prebiotics are integrated, the gut microbiota also shows a promising healthy condition [122]. With regard to the bioactive enzyme functions in the pathogen, recent study identifies a small molecule substance which holds the promise to inhibit the genotoxic effect by binding to the active site [123]. Thus, candidate carcinogenetic microbes will be destroyed without the side-effects of antibiotics. In addition, as well as antibiotic and proton pump inhibition, drugs could also deliver a beneficial effect on the imbalanced gut microbiota of some diseases. Berbelin, an isoquinoline alkaloid used for intestinal infection, has been proved to reverse the increased opportunistic pathogen proportion and inhibit the activation of related carcinogenesis pathways [124].

### Current predicament and future direction

In the former parts of this paper, we mainly describe and discuss current achievements in colorectal cancer. When we perform the experiments, the methods chosen are critically important to our study results. Just as described above, the association study between microbiota and colorectal cancer is sometime based on tumor tissue and matched normal tissue. However, intestinal cleaning is a regular preparation activity for tumorectomy. The pharmaceuticals used will surely have an impact on the gut tissue associated microbiota composition. The use of enema has been confirmed to affect the fecal microbial load, and it was shown that restoration to the previous gut microbiota structure took at least 2 weeks [125]. However, based on the current perspective, the tissue samples obtained during the operation are still the optimized sample for further colorectal cancer study. Another question that needs to be assessed is how to choose the best way to improve our gut microbiota among the options of probiotics, diet, or sports, and which condition of gut microbiota can be considered as the standard condition in the gut. The field of gut microbiota has become a promising new frontier that impacts host health and disease, and is worthy of more attention in studying and application [126]. With the application of cohort and case–control studies in the population by use of multi-omics, as well as studies of model animals and cells (Fig. 2), we believe that the relationship between gut microbiota and colorectal cancer will be explored more deeply and demonstrated more accurately. In conclusion, we need more evidence to support the causality role of gut microbiota in colorectal cancer, and also more clinical practice in the management of colorectal cancer.
